# Supplementary material for: Father–Mother Co-Involvement in Child Maltreatment: Associations of Prior Perpetration, Parental Substance Use, Parental Medical Conditions, Inadequate Housing, and Intimate Partner Violence with Different Maltreatment Types
Source: Children (Basel). 2023 Apr 11;10(4):707. doi: 10.3390/children10040707 (PMC10136478; doi:10.3390/children10040707)
Supplement: Supplementary file 1 [file children-10-00707-s001.zip › children-2315815-supplementary.pdf]

Supplemental Material S1

Table S1. *Logistic Regression Results Predicting Mother-Father Co-Involvement in Physical Abuse and Neglect by Child Sex*

Variables	Physical Abuse		Neglect	
	Girl<br>AOR 95% CI	Boy<br>AOR 95% CI	Girl<br>AOR 95% CI	Boy<br>AOR 95% CI
**Family risk factors**				
Prior perpetrator	**0.57 [0.43, 0.76]**	**0.47 [0.36, 0.61]**	1.89 [1.12, 3.17]	**2.52 [1.47, 4.32]**
Substance use	**0.77 [0.60, 0.99]**	0.82 [0.64, 1.05]	**3.19 [1.72, 5.91]**	**3.16 [1.73, 5.79]**
Mental health problems	0.72 [0.42, 1.22]	0.74 [0.42, 1.28]	2.88 [0.83, 9.39]	0.96 [0.24, 3.80]
Medical complexity	0.91 [0.55, 1.49]	0.85 [0.52,1.40]	0.71 [0.32, 1.57]	0.62 [0.26, 1.51]
Inadequate housing	0.70 [0.49, 1.00]	**0.53 [0.36, 0.77]**	**2.57 [1.31, 5.04]**	**2.38 [1.13, 5.02]**
Economic insecurity	1.01 [0.75, 1.34]	1.25 [0.94, 1.67]	1.10 [0.64, 1.88]	0.89 [0.51, 1.57]
Intimate partner violence	0.95 [0.74, 1.23]	0.78 [0.60, 1.02]	1.59 [0.96, 2.63]	**2.14 [1.23, 3.73]**
**Sociodemographic factors**				
Fathers' age	0.99 [0.98, 1.01]	0.99 [0.97, 1.01]	1.04 [1.00, 1.08]	0.99 [0.96, 1.02]
Mothers' age	**0.97 [0.94, 0.99]**	1.00 [0.98, 1.03]	1.01 [0.96, 1.05]	1.03 [0.98, 1.09]
Couples' race and ethnicity (reference: White):				
Black	1.40 [1.00, 1.97]	1.33 [0.96, 1.84]	0.86 [0.43, 1.75]	**0.49 [0.27, 0.88]**
Latinx	0.83 [0.54, 1.28]	0.87 [0.57, 1.31]	0.93 [0.46, 1.87]	**0.49 [0.24, 0.99]**
Other	1.00 [0.74, 1.36]	1.02 [0.75, 1.39]	1.12 [0.63, 2.00]	1.13 [0.60, 2.13]
Child age	1.00 [0.96, 1.03]	**0.91 [0.87, 0.96]**	**0.93 [0.88, 0.99]**	1.03 [0.96, 1.11]

Notes. AOR = Adjusted Odds Ratio. CI = Confidence Interval. Bolded indicate significant adjusted odds ratios. All models included state dummy variables.

Table S2. *Logistic Regression Results Predicting Mother-Father Co-Involvement in Emotional Abuse and Sexual Abuse by Child Sex*

Variables	Emotional Abuse		Sexual Abuse	
	Girl<br>AOR 95% CI	Boy<br>AOR 95% CI	Girl<br>AOR 95% CI	Boy<br>AOR 95% CI
**Family risk factors**				
Prior perpetrator	0.49 [0.22, 1.10]	0.92 [0.46, 1.89]	0.49 [0.31, 0.80]	0.93 [0.36, 2.45]
Substance use	0.67 [0.30, 1.51]	**0.40 [0.16, 0.99]**	**0.10 [0.04, 0.23]**	0.40 [0.16, 1.04]
Mental health problems	2.31 [0.70, 7.61]	1.03 [0.12, 8.97]	1.94 [0.93, 4.04]	**3.35 [1.06, 10.6]**
Medical complexity	0.32 [0.02, 4.98]	1.23 [0.34, 4.50]	1.50 [0.64, 3.50]	1.98 [0.64, 6.16]
Inadequate housing	0.48 [0.17, 1.36]	0.42 [0.15, 1.13]	0.50 [0.20, 1.30]	0.43 [0.11, 1.72]
Economic insecurity	0.49 [0.21, 1.12]	0.65 [0.32, 1.30]	0.61 [0.32, 1.14]	0.95 [0.34, 2.63]
Intimate partner violence	**4.63 [1.80, 11.9]**	**6.21 [2.69, 14.3]**	0.60 [0.31, 1.13]	0.72 [0.23, 2.24]
**Sociodemographic factors**				
Fathers' age	**0.85 [0.76, 0.96]**	0.98 [0.93, 1.04]	1.00 [0.95, 1.05]	1.00 [0.92, 1.09]
Mothers' age	0.99 [0.88, 1.12]	**0.85 [0.79, 0.91]**	**0.89 [0.84, 0.94]**	**0.82 [0.72, 0.92]**
Couples' race and ethnicity (reference: White):				
Black	0.54 [0.13, 2.28]	1.19 [0.41, 3.44]	**0.31 [0.13, 0.72]**	0.23 [0.03, 1.84]
Latinx	0.20 [0.02, 2.13]	0.46 [0.06, 3.88]	1.54 [0.76, 3.12]	1.00 [1.00, 1.00]
Other	1.43 [0.60, 3.38]	1.19 [0.54, 2.63]	0.46 [0.20, 1.04]	1.65 [0.69, 3.94]
Child age	1.26 [1.15, 1.38]	**1.19 [1.09, 1.30]**	1.35 [1.28, 1.42]	**1.28 [1.16, 1.41]**

Notes. AOR = Adjusted Odds Ratio. CI = Confidence Interval. Bolded indicate significant adjusted odds ratios. All models included state dummy variables.

Supplemental Material S2

Table S3. *Logistic Regression Results Predicting Mother-Father Co-Involvement in Physical Abuse by Child Age*

Variables	Infancy/Toddlerhood AOR 95% CI	Early Childhood AOR 95% CI	School Age AOR 95% CI	Adolescence AOR 95% CI
**Family risk factors**				
Prior perpetrator	**0.44 [0.35, 0.55]**	0.78 [0.45, 1.35]	**0.46 [0.25, 0.87]**	**0.98 [0.46, 2.08]**
Substance use	1.00 [0.81, 1.22]	0.66 [0.37, 1.18]	**0.32 [0.16, 0.65]**	**0.39 [0.17, 0.86]**
Mental health problems	0.72 [0.45, 1.15]	0.53 [0.20, 1.40]	1.44 [0.50, 4.15]	0.56 [0.16, 1.96]
Medical complexity	0.82 [0.53, 1.25]	2.07 [0.87, 4.89]	0.56 [0.19, 1.67]	0.57 [0.11, 2.96]
Inadequate housing	**0.53 [0.38, 0.74]**	0.56 [0.27, 1.15]	0.77 [0.35, 1.71]	1.47 [0.52, 4.12]
Economic insecurity	0.99 [0.78, 1.26]	1.17 [0.59, 2.34]	1.44 [0.70, 2.95]	2.18 [0.95, 5.01]
Intimate partner violence	**0.78 [0.62, 0.98]**	1.06 [0.58, 1.95]	1.24 [0.68, 2.27]	1.40 [0.62, 3.16]
**Sociodemographic factors**				
Fathers' age	0.99 [0.98, 1.01]	0.96 [0.92, 1.01]	0.96 [0.91, 1.01]	0.99 [0.93, 1.05]
Mothers' age		0.99 [0.93, 1.05]		0.95 [0.89, 1.02]
Couples' race and ethnicity (reference: White):				
Black	1.21 [0.91, 1.60]	1.30 [0.61, 2.75]	1.63 [0.79, 3.36]	1.90 [0.90, 4.00]
Latinx	1.02 [0.73, 1.44]	**0.19 [0.04, 0.90]**	0.34 [0.11, 1.07]	1.03 [0.32, 3.35]
Other	0.99 [0.76, 1.28]	1.41 [0.71, 2.81]	1.10 [0.51, 2.38]	0.94 [0.31, 2.80]
Child sex (female)	0.83 [0.69, 1.00]	1.22 [0.72, 2.06]	0.84 [0.52, 1.36]	1.58 [0.88, 2.87]

Notes. AOR = Adjusted Odds Ratio. CI = Confidence Interval. Bolded indicate significant adjusted odds ratios. All models included state dummy variables.

Table S4. *Logistic Regression Results Predicting Mother-Father Co-Involvement in Neglect by Child Age*

Variables	Infancy/Toddlerhood AOR 95% CI	Early Childhood AOR 95% CI	School Age AOR 95% CI	Adolescence AOR 95% CI
**Family risk factors**				
Prior perpetrator	**2.43 [1.55, 3.79]**	1.62 [0.53, 4.93]	3.67 [1.16, 11.6]	0.68 [0.20, 2.34]
Substance use	**2.74 [1.76, 4.28]**	4.69 [0.66, 33.4]	2.36 [0.60, 9.31]	3.40 [0.75, 15.4]
Mental health problems	1.26 [0.46, 3.41]	1.00 [1.00, 1.00]	0.36 [0.09, 1.47]	1.00 [1.00, 1.00]
Medical complexity	1.05 [0.45, 2.45]	1.00 [1.00, 1.00]	0.27 [0.07, 1.05]	0.57 [0.10, 3.37]
Inadequate housing	**2.15 [1.16, 3.98]**	3.00 [0.72,12.6]	2.11 [0.47, 9.41]	2.78 [0.59, 13.0]
Economic insecurity	1.03 [0.63, 1.68]	0.75 [0.20, 2.79]	0.46 [0.13, 1.61]	2.36 [0.64, 8.71]
Intimate partner violence	1.54 [1.01, 2.34]	2.34 [0.53, 10.3]	**4.70 [1.13, 19.6]**	0.80 [0.20, 3.26]
**Sociodemographic factors**				
Fathers' age	1.01 [0.98, 1.03]	1.12 [0.99, 1.27]	1.02 [0.91, 1.14]	1.04 [0.95, 1.13]
Mothers' age	1.03 [0.99, 1.08]	1.00 [0.85, 1.18]	1.08 [0.96, 1.21]	1.05 [0.96, 1.15]
Couples' race and ethnicity (reference: White):				
Black	0.66 [0.39, 1.12]	2.86 [0.58, 14.2]	0.42 [0.19, 1.51]	0.23 [0.05, 1.11]
Latinx	0.61 [0.33, 1.10]	0.19 [0.03, 1.35]	3.17 [0.34, 29.5]	0.71 [0.16, 3.27]
Other	1.58 [0.95, 2.65]	0.32 [0.09, 1.15]	0.65 [0.27, 2.01]	0.53 [0.05, 5.68]
Child sex (female)	0.96 [0.66, 1.38]	1.30 [0.50, 3.35]	1.33 [0.55, 3.38]	0.46 [0.20, 1.03]

Notes. AOR = Adjusted Odds Ratio. CI = Confidence Interval. Bolded indicate significant adjusted odds ratios. All models included state dummy variables.
